# YouTube Videos as a Source of Information About Immunology for Medical Students: Cross-Sectional Study

**DOI:** 10.2196/12605

**Published:** 2019-05-28

**Authors:** Jef Van den Eynde, Alexander Crauwels, Philip Georg Demaerel, Lisa Van Eycken, Dominique Bullens, Rik Schrijvers, Jaan Toelen

**Affiliations:** 1 Faculty of Medicine KU Leuven Leuven Belgium; 2 Department of Microbiology and Immunology KU Leuven Leuven Belgium; 3 Division of Woman and Child Department of Pediatrics UZ Leuven Leuven Belgium; 4 Division of Allergy and Clinical Immunology Department of General Internal Medicine UZ Leuven Leuven Belgium; 5 Department of Development and Regeneration KU Leuven Leuven Belgium

**Keywords:** antigen presentation, education, immunoglobulins, immunology, learning, students

## Abstract

**Background:**

The use of the internet as a source of information has grown exponentially in the last decade. YouTube is currently the second most visited website and a major Web-based educational resource for medical students.

**Objective:**

The aim of this study was to evaluate the quality, accuracy, and attractiveness of the information acquired from YouTube videos about 2 central concepts in immunology.

**Methods:**

YouTube videos posted before August 27, 2018 were searched using selected keywords related to either antigen presentation or immunoglobulin gene rearrangement. Video characteristics were recorded, and the Video Power Index (VPI) was calculated. Videos were assessed using 5 validated scoring systems: understandability and attractiveness, reliability, content and comprehensiveness, global quality score (GQS), and a subjective score. Videos were categorized by educational usefulness and by source.

**Results:**

A total of 82 videos about antigen presentation and 70 about immunoglobulin gene rearrangement were analyzed. Videos had a mean understandability and attractiveness score of 6.57/8 and 5.84/8, content and comprehensiveness score of 9.84/20 and 5.84/20, reliability score of 1.65/4 and 1.53/4, GQS of 3.38/5 and 2.76/5, and subjective score of 2.00/3 and 2.00/3, respectively. The organized channels group tended to have the highest VPI and GQS.

**Conclusions:**

YouTube can provide medical students with some useful information about immunology, although content wise it cannot substitute textbooks and academic courses. Students and teachers should be aware of the educational quality of available videos if they intend to use them in the context of blended learning.

## Introduction

### Background

The use of the internet as a source of both general and specific information has grown exponentially over the years, with an estimated 54.4% of the world population having access to internet in 2018 [1]. Some of its major assets are the ease and efficiency with which new knowledge can be acquired. For this reason, the internet has also gained popularity among medical students and subsequently changed the way they learn [2,3]. Currently, 94% of medical students are actively participating in social media applications, compared with 79% of residents and 42% of physicians [4]. A recent review shows that university students spend increasingly more time on the internet for educational purposes [5]. This evolution is promoted by the numerous advantages of the internet, such as ease of access and adaptability to individual timetables and can lead to an increase in academic performance [5,6]. However, concerns have been raised about the accuracy and reliability of the available information on the Web [7-10].

Much of the information that medical students have to process is abstract and often requires visual representation for perfect understanding (eg, immunoglobulin gene rearrangement). YouTube, characterized by its audiovisual material, has become a major auxiliary source of information for students often complementary with their textbooks and academic courses [11]. YouTube is currently the second most visited website on the internet and has over 1 billion users, which equals almost one-third of the internet users [12,13]. Once created for entertainment purposes, the site nowadays also contains educational videos posted by individuals, professionals, organizations, and companies [13,14]. The content is accrescent, with an upload rate of 300 hours per minute and a watch rate of 5 billion videos per day [15].

There are, however, no checks and balances, and videos do not have to undergo the same review process as the publication of journal articles and textbooks, which results in variable content quality and uncertainty about sources and reliability [16]. At the same time, medical students may not always be able to accurately recognize ambiguous information [17]. Research is therefore needed to assess the reliability and accuracy of information presented in educational videos. This has already been conducted for some medical-related topics such as anatomy [11], pharmacokinetics [18], and physical examination [19].

### Objectives

Immunology is an exemplary topic for the use of educational videos, as it contains multiple abstract concepts that benefit from a visual representation of complex physiological processes. YouTube provides the advantage of giving audiovisual information in a very accessible manner, but the quality of educational videos related to the field of immunology has not been investigated. This study focused on videos about 2 exemplary concepts that are part of the core curriculum in clinical immunology and are generally considered challenging by students: antigen presentation and immunoglobulin gene rearrangement. The aim was to assess the quality, accuracy, and attractiveness of these videos and to determine if YouTube can be a useful source of information for medical students.

## Methods

### Search Strategy

The search engine of YouTube was queried for 2 different subjects related to immunology: antigen presentation and immunoglobulin gene rearrangement. Videos about antigen presentation were searched using the keywords *MHC 1, MHC 2, MHC I, MHC II, MHC 1 and 2, MHC I and II, Antigen presentation, cross presentation, HLA class I,* and *HLA class II*. Videos about immunoglobulin gene rearrangement were searched using the keywords *VDJ recombination, immunoglobulin gene rearrangement, antibody diversity genetics, immunoglobulin heavy chain genetics, immunoglobulin variable region genetics, organization and expression of immunoglobulin genes, immunoglobulin genetics,* and *immunoglobulin gene organization.* These terms were optimized using a snowballing technique based on the sequential suggestions of the autofill function of YouTube and Medical Subject Headings terms of PubMed articles related to the subjects. Each term was searched in a separate YouTube search window on August 27, 2018 via the default settings and in an incognito browser window. The first 60 results for each term were considered, which has been shown to correspond to the amount of videos internet users usually screen [20]. Videos were excluded if they were irrelevant, the duration exceeded 60 min, the target audience was not students, the language was not English, or the video contained advertising. This search method simulates the actual search strategy of students. After exclusion, 82 videos about antigen presentation and 70 videos about immunoglobulin gene rearrangement were selected for analysis.

In addition, a cross-section of overall YouTube videos and biology-related YouTube videos was made using the pages *Entertainment – Topic* and *Biology - Topic*, respectively. These are pages that are autogenerated by YouTube and collect videos that have content related to a specific topic. Videos were registered based on similar exclusion criteria as described above (except specific target audience) until 100 videos for each list were collected.

### Data Collection

The following characteristics of the YouTube videos were recorded: number of views, number of likes and dislikes, number of comments, duration, year of publication, and days since upload. The like ratio (like*100/[like+dislike]), view ratio (number of views/days), and Video Power Index (VPI; like ratio*view ratio/100) were also determined.

The videos were categorized into groups based on their source and educational usefulness. Categories based on the source were as follows: (1) student (authors were students posting individual videos), (2) organized channel (organized YouTube channels by tutors, teachers, or professors dedicated to producing educational videos), and (3) other (videos from textbooks and audiobooks, which have passed a review procedure). Categories based on educational usefulness were a function of the Global Quality Scale (GQS) and categorized as “useful” (GQS>3) or “not useful” (GQS≤3), with “useful videos” being considered as those videos that contribute in a reasonable extent to the student’s knowledge and can be advised as qualitative learning material.

### Video Evaluation

All videos were independently evaluated by 2 researchers for each subject (JVDE and PGD for antigen presentation and LVE and AC for immunoglobulin gene rearrangement, respectively) using 5 different scoring systems (see Multimedia Appendices 1-5). All videos were evaluated by medical students that had already attended the immunology course in their curriculum.

Understandability and attractiveness (U&A) was scored using an 8-point modified Patient Education Materials Tool (PEMAT) score, which was adapted for a medical student perspective from the original PEMAT tool for the assessment of audio-visual patient information by Shoemaker et al [21]. The 4-point reliability scoring was based on the Journal of American Medical Association benchmark criteria for reliability and accuracy [16,22]. The content and comprehensiveness scorings (C&C) were composed by using recent review articles from high-impact journals: a 22-point score for antigen presentation [23,24] and a 26-point score for immunoglobulin gene rearrangement [25,26], which were both normalized to a 20-point score for statistical analysis and data representation. The overall quality of each video was rated using the 5-point GQS, which was developed as an evaluation tool for the assessment of the flow and ease of use of information on health-related websites [14,16,27]. Finally, a subjective score was attributed to the videos according to the pleasantness of watching them, consisting of 3 points: (1) unpleasant to watch, (2) pleasant to watch, and (3) very pleasant to watch. Videos with different scorings were reassessed until a consensus was reached.

### Statistical Analyses

The Shapiro-Wilk test was used to evaluate normality of data. Continuous variables are expressed as mean (95% CI), and an intergroup comparison was carried out using the nonparametric Kruskal-Wallis test or parametric 1-way analysis of variance according to data distribution. Respectively, Dunn test or Fisher least significant difference test were used as a post hoc test for multiple comparison if 3 groups were compared; Ryan-Einot-Gabriel-Welsch and Quiot test was used as a post hoc test when more than 3 groups were compared. Categorical variables are expressed as frequency and proportion, and differences were assessed with the chi-square test. Spearman rho correlation analysis was used to assess correlations between parameters. Stepwise multiple linear regression models were constructed to predict C&C score based on audience interaction parameters and video source. All tests were 2-sided, and a *P* value less than .05 was deemed statistically significant. All analyses have been performed using SPSS software (SPSS Inc).

## Results

### Audience Interaction Parameters

**Figure 1:**
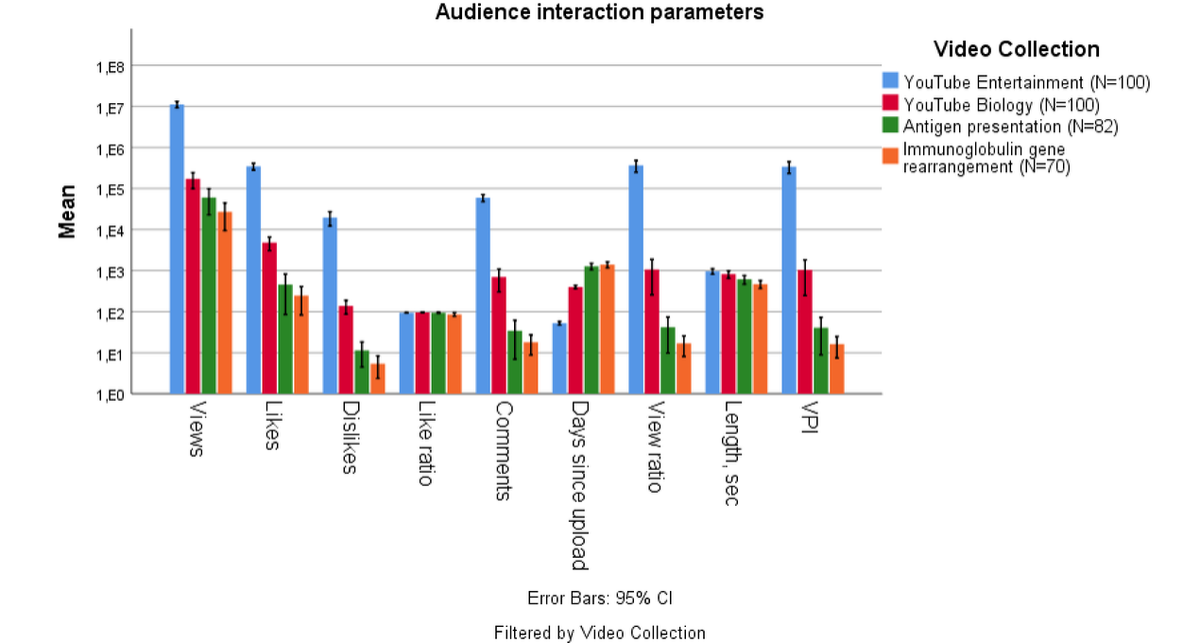
Audience interaction parameters. Values in the Y-axis are given as mean and are provided with 95% CIs. VPI: Video Power Index.

The mean audience interaction parameters of overall YouTube videos, biology-educational videos, and the videos about antigen presentation and immunoglobulin gene rearrangement are represented in Figure 1. Numerical data and pairwise comparisons are given in Multimedia Appendices 6 and 7, respectively. Both immunological videos had equal audience interaction parameters. Overall, YouTube videos were substantially more popular than all other videos, and the educational YouTube videos also showed higher audience interaction parameters than the immunological videos in our analysis.

Of the 82 videos about antigen presentation, 28.05% (23/82) were categorized as *student*, 59.76% (49/82) as *organized channel*, and 12.20% (10/82) as *other* (Table 1 and Multimedia Appendix 8). There was a statistically significant difference in views, likes, dislikes, comments, days since upload, view ratio, length of video, and VPI. When pairwise comparisons were made, views, likes, dislikes, comments, view ratio, and VPI were significantly higher in the *organized channel* videos than in the *student* videos but not different from the *other* videos. *Other* videos had significantly more days since upload when compared with *student* videos but had a shorter duration when compared with *organized channel* videos. Videos were also categorized by educational usefulness, with 42.68% (35/82) of the videos classified as *useful* and 57.32% (47/82) as *not useful* (Table 2). Videos classified as not useful had significantly more days since upload and had a shorter length. Organized channels were most frequently categorized as useful, with 77.1% (27/35) of this source contributing to the useful videos.

**Table 1:** Antigen presentation videos: categorized by source.

Variable		Student	Organized channel	Other	*P* value
Video number, n (%)		23 (28.1)	49 (59.8)	10 (12.2)	—^a^
**Audience interaction parameters, mean (95% CI)**					
	Views^b^	8.2 (-1.2 to 17.6)	71.6 (17.6 to 125)^c^	70.1 (7.2 to 133)	.005^d^
	Likes	35.4 (-0.09 to 70.9)	634 (66.3 to 1201)^c^	230 (3.6 to 456)	.01^d^
	Dislikes	9.9 (-8.4 to 28.1)	11.6 (4.6 to 18.5)^c^	5.8 (1.4 to 10.2)	.01^d^
	Like ratio	95.6 (91.3 to 100)	94.1 (89.4 to 98.8)	91.6 (79.4 to 104)	.31
	Comments	2.6 (-0.05 to 5.2)	47.6 (7.5 to 87.6)^c^	11.0 (1.1 to 20.9)	.002^d^
	Days since upload	959 (599 to 1318)	1168 (937 to 1399)	2252 (1312 to 3191)^c^	.02^d^
	View ratio	6.5 (-0.9 to 13.9)	54.7 (7.7 to 102)^c^	22.2 (3.3 to 41.0)	.001^d^
	Length, seconds	481 (306 to 656)	700 (512 to 887)	380 (-87.2 to 847)^e^	.003^d^
	Video Power Index	6.0 (-0.3 to 12.2)	57.8 (7.3 to 108)^c^	23.8 (3.6 to 44.0)	.007^d^
**Content, mean (95% CI)**					
	Reliability	1.4 (1.05-1.7)	1.7 (1.5-1.8)	2.2 (1.5-2.9)^c^	.01^d^
	Content and comprehensiveness	8.3 (6.4-10.3)	11.0 (9.62-12.27)	7.9 (5.0-10.9)	.046^d^
	Global quality score	3.0 (2.6-3.4)	3.7 (3.4-3.9)^c^	2.8 (2.2-3.4)^e^	.001^d^
**Cinematography, mean (95% CI)**					
	Understandability and attractiveness	6.1 (5.6-6.7)	6.9 (6.6-7.2)	6.0 (4.5-7.5)	.06
	Subjective score	1.9 (1.6-2.2)	2.08 (1.9-2.3)	1.9 (1.3-2.5)	.47

^a^Not applicable.

^b^These factors were divided by 1000.

^c^*P*<.05 versus *student*.

^d^*P*<.05 was considered significant.

^e^*P*<.05 versus *organized channel*.

**Table 2:** Antigen presentation videos: categorized by educational usefulness.

Variable		Useful	Not useful	*P* value
Video number, n (%)		35 (42.7)	47 (57.3)	—^a^
Global Quality Score, mean (95% CI)		4.3 (4.1-4.4)	2.7 (2.6-2.8)	—
**Audience interaction parameters, mean (95% CI)**				
	Views^b^	30.7 (12.9-48.5)	70.7 (14.0-127.3)	.91
	Likes	340 (140-541)	470 (-104-1044)	.18
	Dislikes	7.4 (1.8-12.9)	12.5 (2.3-22.7)	.99
	Like ratio	97.7 (96.6-98.9)	91.3 (85.5-97.2)	.23
	Comments	28.03 (9.2-46.8)	32.3 (-8.08-72.7)	.20
	Days since upload	969 (703-1235)	1445 (1141-1748)	.04^c^
	View ratio	24.9 (11.9-37.9)	46.4 (-2.6-95.3)	.31
	Length, seconds	922 (662-1181)	359 (278-440)	<.001^c^
	Video Power Index	25.9 (12.1-39.6)	52.4 (-4.5-109)	.63
**Content**				
	Reliability, mean (95% CI)	1.7 (1.5-1.9)	1.6 (1.4-1.9)	.17
	Content and comprehensiveness, median (IQR)^d^	15 (13-19)	7 (6-10)	<.001^c^
**Cinematography, mean (95% CI)**				
	Understandability and attractiveness	7.0 (6.7-7.4)	6.3 (5.8-6.7)	.02^c^
	Subjective score	2.3 (2.07-2.5)	1.8 (1.6-2.01)	.002^c^
**Source, n (%)**				
	Student	6.0 (17.1)	17.0 (36.2)	.02^c^
	Organized channel	27.0 (77.1)	22.0 (46.8)	—
	Other	2.0 (5.7)	8.0 (17.0)	—

^a^Not applicable.

^b^These factors were divided by 1000.

^c^*P*<.05 was considered significant.

^d^IQR: interquartile range.

Of the 70 videos about immunoglobulin gene rearrangement, 14.29% (10/70) were categorized as *student*, 51.43% (36/70) as *organized channel*, and 34.29% (24/70) as *other*. There was a statistically significant difference in days since upload and length between these groups (Table 3 and Multimedia Appendix 9). *Student* videos were observed to have significantly more days since upload than those of the other groups, and *organized channel* videos had a significantly longer duration. Videos were also categorized by educational usefulness, with 24.29% (17/70) videos classified as *useful* and 75.71% (53/70) as *not useful* (Table 4). Videos classified as not useful had a significantly lower number of views, dislikes, and comments. They also had a lower view ratio and a shorter length. There was no source that was categorized significantly more frequently as useful.

**Table 3:** Immunoglobulin gene rearrangement videos: categorized by source.

Variable		Student	Organized channel	Other	*P* value
Video number, n (%)		10 (14.3)	36 (51.4)	24 (34.3)	—^a^
**Audience interaction parameters, mean (95% CI)**					
	Views^b^	4.9 (–1.6 to 11.4)	35.0 (10.7 to 59.3)	11.2 (–2.09 to 24.4)	.76
	Likes	43.9 (–38.06 to 126)	304 (54.9 to 553)	103 (–15.4 to 221)	.48
	Dislikes	1.3 (–1.4 to 3.9)	4.1 (1.3 to 6.9)	5.5 (–0.03 to 11.03)	.28
	Like ratio	98.4 (95.4 to 101)	90.5 (80.5 to 100)	74.8 (55.4 to 94.2)	.06
	Comments	6.2 (–4.1 to 16.5)	22.8 (9.4 to 36.1)	5.04 (0.4 to 9.7)	.47
	Days since upload	1907 (1599 to 2215)	1199 (918 to 1480)^c^	1027 (668 to 1386)^b^	.02^d^
	View ratio	2.3 (–0.4 to 5.03)	17.9 (6.2 to 29.6)	12.04 (3.2 to 20.9)	.42
	Length, seconds	432 (257 to 608)	578 (446 to 711)	218 (128 to 307)^d^	<.001^d^
	Video Power Index	2.3 (–1.08 to 5.7)	22.7 (5.9 to 39.4)	13.8 (2.9 to 24.8)	.24
**Content, mean (95% CI)**					
	Reliability	0.9 (0.7 to 1.1)	1.9 (1.6 to 2.2)^c^	1.3 (1.03 to 1.6)^d^	.001^d^
	Content and comprehensiveness	5.2 (2.4 to 7.9)	6.6 (5.1 to 8.07)	5.0 (3.09 to 6.9)^d^	.11
	Global quality score	2.8 (2.06 to 3.5)	3.08 (2.7 to 3.4)	2.3 (1.8 to 2.7)	.02^d^
**Cinematography, mean (95% CI)**					
	Understandability and attractiveness	5.9 (5.1 to 6.7)	6.1 (5.6 to 6.6)	5.4 (4.6 to 6.1)	.23
	Subjective score	1.9 (1.5 to 2.3)	2.2 (1.9 to 2.5)	1.8 (1.4 to 2.09)	.10

^a^Not applicable.

^b^These factors were divided by 1000.

^c^*P*<.05 versus *student*.

^d^*P*<.05 was considered significant.

^e^*P*<.05 versus *organized channel*.

**Table 4:** Immunoglobulin gene rearrangement videos: categorized by educational usefulness.

Variable		Useful	Not useful	*P* value
Video number, n (%)		17 (24.3)	53 (75.7)	—^a^
Global quality score, mean (95% CI)		4.2 (4.01-4.5)	2.3 (2.07-2.5)	—
**Audience interaction parameters, mean (95% CI)**				
	Views^b^	53.2 (5.2-101.2)	12.7 (3.8-21.6)	.03^c^
	Likes	527 (6.5-1049)	94.6 (24.0-165)	.10
	Dislikes	7.1 (1.5-12.8)	3.4 (0.7-6.2)	.05^c^
	Like ratio	84.4 (63.7-105)	86.5 (76.9-96.0)	.77
	Comments	34.8 (10.9-58.7)	7.2 (2.5-11.9)	.002^c^
	Days since upload	1272 (814-1730)	1231 (1004-1458)	.94
	View ratio	26.7 (4.4-49.05)	9.5 (4.04-14.9)	.009^c^
	Length, seconds	812 (618-1007)	313 (243-382)	<.001^c^
	Video Power Index	29.7 (2.7-56.8)	11.05 (4.0-18.1)	.22
**Content**				
	Reliability, mean (95% CI)	1.89 (1.5-2.3)	1.4 (1.2-1.6)	.03^c^
	Content and comprehensiveness, median (IQR)^d^	12 (10.5-21)	5 (3-7)	<.001^c^
**Cinematography, mean (95% CI)**				
	Understandability and attractiveness	6.8 (6.3-7.4)	5.5 (5.08-6.0)	.001^c^
	Subjective score	2.5 (2.2-2.8)	1.9 (1.6-2.07)	.005^c^
**Source, n (%)**				
	Student	1.0 (5.9)	9.0 (17.0)	.06
	Organized channel	13.0 (76.5)	23.0 (43.4)	—
	Other	3.0 (17.6)	21.0 (29.6)	—

^a^Not applicable.

^b^These factors were divided by 1000.

^c^*P*<.05 was considered significant.

^d^IQR: interquartile range.

### Content Analysis

The videos about antigen presentation had a mean reliability score of 1.65/4 (SD 0.760), C&C score of 9.84/20 (SD 4.653), and GQS of 3.38/5 (SD 0.911). Between the different groups based on source, there was a significant difference in reliability and C&C (Table 1). When pairwise comparisons were made, the *organized channel* videos had a significantly higher GQS than the 2 other groups, whereas *other* videos had a significantly higher reliability score. C&C and GQS were observed to be higher in *useful* videos (Table 2).

The videos about immunoglobulin gene rearrangement had a mean reliability score of 1.53/4 (SD 0.812), C&C score of 5.84/20 (SD 4.331), and GQS of 2.76/5 (SD 1.096). There was a statistically significant difference in reliability and GQS between groups based on source (Table 3). *Organized channel* videos had a significantly higher GQS than *other* videos and had the highest reliability score of all of the groups. C&C and GQS were observed to be higher in *useful* videos (Table 4).

### Cinematographic Analysis

The videos about antigen presentation had a mean U&A score of 6.57/8 (SD 1.334) and subjective score of 2.00/3 (SD 0.737). There were no significant differences regarding cinematographic scorings between groups based on source (Table 1). U&A and subjective score were observed to be higher in videos classified as useful (Table 2).

The videos about immunoglobulin gene rearrangement had a mean U&A score of 5.84/8 (SD 1.585) and subjective score of 2.00/3 (SD 0.799). When comparing videos based on source, there were no significant differences regarding cinematographic scorings (Table 3). U&A and subjective score were observed to be higher in useful videos (Table 4).

### Stepwise Multiple Linear Regression

The stepwise multiple linear regression analysis for the videos about antigen presentation revealed a significant regression equation (*F*_2,77_=27.591; *P*<.001), with an R^2^ of 0.264. The predicted C&C score is equal to 8.190 + 0.004 (length in seconds). The C&C score increased 0.004 points for each second a video lasted longer. All other audience interaction parameters and video source were excluded from the model as they were no significant predictors.

In the immunoglobulin gene rearrangement group, a significant regression equation with an R^2^ of 0.407 was found (*F*_1,62_=42.547; *P*<.001). The predicted C&C score is equal to 2.987 + 0.010 (length in seconds). C&C score increased 0.010 points for each second a video lasted longer. All other audience interaction parameters and video source were excluded from the model as they were no significant predictors.

The correlation between different scoring systems are given in Multimedia Appendices 10 and 11.

## Discussion

### Principal Findings

The main reason for this research was the assessment of YouTube as a Web-based educational source for medical students on major concepts in clinical immunology [12-14]. Due to its rich audiovisual content, the site is especially attractive in the context of blended learning where traditional classroom methods are combined with Web-based digital media. It enables students to control aspects of individual learning such as time, place, or pace. The availability of audiovisual educational material is especially important for the study of (patho)physiological processes that involve multiple regulators, complex sequences, and feedback loops. Such concepts may be difficult to comprehend for novices when they have to rely solely on a single presentation in the classroom and individual study using textbooks or review articles [11]. High-quality Web-based videos could facilitate the understanding of these subjects, especially if the local institution does not provide access to audiovisual educational material [28,29]. However, the publication of YouTube videos is not automatically subjected to the scrutiny of the classical scientific review process or the guarantee of the academic expert, resulting in variable quality and sometimes misleading information, which might not always be detected by students [16,17].

In this study, we selected 2 important immunological concepts where a visual representation of all the sequential and interacting processes is imperative for understanding. Both antigen presentation and immunoglobulin gene rearrangement are ideal *exemplar* concepts as they are part of the core curriculum in clinical immunology, are well defined, well studied, and not too novel to be present in Web-based educational videos. In our study, we selected movies based on keywords that medical students would use to search for relevant videos on these 2 topics. For every keyword, we scanned the top 60 videos as this is the maximum amount of hit the average internet user screens during a Web search [20]. After the final selection, we assessed content, cinematography, and demographic data of these movies by means of validated scoring systems. A total of 3 of our scoring systems quantify the scientific content either very objectively (reliability score and C&C, using a *gold standard*) or in a more subjective manner (GQS). We found that videos on both topics had mean C&C scores between 5.8 and 9.8 out of a total 20, reliability scores of 1.5 to 1.7 out of 3, and GQS of 2.8 to 3.4 out of 4. This suggests that YouTube videos about 2 central concepts in immunology are generally only of moderate quality and often provide insufficient information for a full “academic” understanding. These results are in line with what has been shown in previous research on medical-related YouTube videos [11,18,19,30,31]. This leads us to corroborate the hypothesis that YouTube resources should be dealt with carefully and should be subjected to critical assessments of accuracy and reliability.

We classified the selected videos by source, as we intended to see if videos made by academic experts scored better when compared with those made by students or other sources. More than half of the videos turned out to be made by *organized channels* (59.76% (49/82) for antigen presentation and 51.43% (36/70) for immunoglobulin gene rearrangement). Contrary to what we anticipated, these videos did not have a higher content score (C&C) or attractiveness score (U&A) compared with the videos of student origin or *other* sources.

In our selection, 3 recordings of academic lectures were found. These had a mean C&C of 17.0/20, a U&A of 7.3/8, a reliability score of 2.3/4, and a GQS of 4.3/5. These scores are significantly higher than those of the overall group, especially with regard to scientific content. These data are in line with other studies reporting that medical educational videos uploaded by universities and research institutes usually score higher on quality scales [18,19]. In sharp contrast to their clearly appropriate and verified content, the academic lecture videos in this study had a markedly lower VPI than the mean of all reviewed videos (4.3 vs 16.2). This illustrates what has already been reported by Desai et al [32] that the most qualitative videos often do not receive the most views.

Interestingly, when the data from the 2 biggest YouTube channels about antigen presentation in our analysis (Armando Hasudungan and Shomu’s Biology) were considered together, a mean C&C of 12.9/20, an U&A of 7.3/8, a reliability score of 1.6/3, and a GQS of 4.1/5 were observed. At the same time, these videos had a relatively high VPI (49.9). This suggests that greatly organized channels dedicated to the creation of high-quality educational videos provide the best Web-based resources for students and that they also are currently being used by these students in larger numbers. To engage students, videos should be appealing and illustrative [33,34]. This was also the case in these videos, which contained beautiful drawings of the pathways involved, making them at once entertaining to watch, something which is reflected in their subjective score of 3/3.

In our study, videos classified as *useful* were found to have higher U&A, C&C, GQS, and subjective scores. In the immunoglobulin gene rearrangement group, a higher reliability score was also observed in these videos as well as a higher number of views. This either suggests that YouTube users might judge about the quality of the information themselves and prefer to watch the more accurate and reliable videos or that the YouTube algorithm preferentially shortlists these videos based on these keywords. The latter is less probable as no significant difference regarding audience interaction parameters was seen in the antigen presentation group, which corresponds with the finding in previous studies assessing YouTube videos, namely that no relationship was observed between number of views, likes, or comments and usefulness [18,19,30]. In general, the majority of the videos from each source were classified as *not useful*.

On the basis of the findings in this study, a preferential search strategy to detect videos with the best and most comprehensive content can be derived. First of all, videos from *organized channel* sources tend to have a higher C&C score than the other sources and were more often classified as *useful*. Students could actively look for such organized channels. Furthermore, the multiple logistic regression model showed that the length of the video was the single most important predictor of the C&C score. It is clear that a relevant overview of these processes cannot be given in a video with a duration of 3 min. The age of the video did not influence the C&C score, most likely because the essentials of these immunological processes did not significantly change in recent years. Views, likes, and comments did not predict C&C score, probably because the search results also included several popular videos that were lacking adequate information or were meant for a lay audience. If students select their video using a relevant keyword and subsequently use the criteria of source and longer duration of the video, they will be more likely to find relevant educational videos on YouTube. Unfortunately, this strategy will never be foolproof. As an illustration, we listed the top 3 and bottom 3 videos (based on C&C score) for videos about antigen presentation and immunoglobulin gene rearrangement (see Tables 5 and 6). In the latter one, 1 of the top 3 videos is provided by a biotechnological company with a special interest in antibody technology rather than an academic institution.

### Limitations

There are some limitations in our study, though. First, we only considered English-language YouTube videos showing up in the first 60 results of each search term but did not check links to videos from other educational websites or videos that were referred to within these videos. However, it has been demonstrated that 90% of all search engine users only click on results within the first 3 pages, which corresponds to our 60 YouTube results [20]. After all, our purpose was to simulate a regular search for educational resources, as would be conducted by a medical student. Furthermore, as 30 seconds of YouTube watching counts as a *view* [47], we were unable to determine whether users were engaged for the full duration of the video. Third, one could imagine that videos that were liked and viewed more in the past are also more likely to be liked and viewed in the future, thereby representing a self-enforcing feedback mechanism. However, this also corresponds to the way students will find and interact with videos during their search. A final limitation is inherent to any research assessing the quality of YouTube videos: we could only cover a single snapshot. YouTube is a dynamic and ever-growing website, and as a consequence, rankings and views are subject to change, with new videos uploaded at a very high pace.

**Table 5:** Top 3 and bottom 3 based on content and comprehensiveness for videos about antigen processing.

Video name		Source	Reference
**Top 3**			
	Immunology Lecture Mini-Course, 4 of 14: Antigen Presentation to T lymphocytes	Albert Einstein College of Medicine	[35]
	Major histocompatibility complex	Shomu's Biology	[36]
	Mod-11 Lec-24 The Major Histocompatibility Complex: MHC class II pathway	NPTEL hrd	[37]
**Bottom 3**			
	MHC Class I	CR King	[38]
	MHC Class I Processing	Garland Science	[39]
	Immune system: MHC proteins	Walter Jahn	[40]

**Table 6:** Top 3 and bottom 3 based on content and comprehensiveness for videos about immunoglobulin gene rearrangement.

Video name		Source	Reference
**Top 3**			
	Models of immunoglobulin gene structure	Vidya-mitra	[41]
	Immunology – antibody somatic (VDJ) recombination II	Armando Hasudungan	[42]
	Antibody diversity rearragment	Creative Biolabs	[43]
**Bottom 3**			
	Where does VDJ recombination occur?	Atunakai3a	[44]
	Medical vocabulary: what does V (D) J recombination mean?	Botcaster inc. bot	[45]
	“Immunology,” Immunoglobulin genes are rearranged in antibody producing cells	MyCyberCollege	[46]

### Conclusions

In conclusion, this study focusing on immunology videos showed that YouTube can provide useful auxiliary resources for students, although it cannot substitute excellent academic lectures, validated textbooks, or state-of-the-art reviews when it comes to scientific accurateness. As most videos were found not to be educationally useful and references or bibliography were often missing, strong critical assessment skills remain imperative if these videos are used as complementary study material. Another important finding was that organized YouTube channels dedicated to Web-based educational videos provide the most qualitative and appealing resources. Students and educators alike should be aware of the quality of available videos, and increasing effort should be spent on collecting videos suited for medical students on channels with appealing, reliable, and accurate information.

## Appendix

Multimedia Appendix 1 Scoring system: Understandability and attractiveness.

Multimedia Appendix 2 Scoring system: Reliability.

Multimedia Appendix 3 Scoring system: Content and comprehensiveness, antigen presentation.

Multimedia Appendix 4 Scoring system: Content and comprehensiveness, immunoglobulin gene rearrangement.

Multimedia Appendix 5 Scoring system: Global Quality Score.

Multimedia Appendix 6 Audience interaction parameters.

Multimedia Appendix 7 Audience interaction parameters, pairwise comparison.

Multimedia Appendix 8 Antigen presentation videos: categorized by source, pairwise comparison.

Multimedia Appendix 9 Immunoglobulin gene rearrangement videos: categorized by source, pairwise comparison.

Multimedia Appendix 10 Correlations between video scoring systems in the antigen presentation group.

Multimedia Appendix 11 Correlations between video scoring systems in the immunoglobulin gene rearrangement group.

## Abbreviations

C&C: content and comprehensiveness score
GQS: Global Quality Score
PEMAT: Patient Education Materials Tool
U&A: understandability and attractiveness score
VPI: Video Power Index
