# Supplementary material for: YouTube Videos as a Source of Information About Immunology for Medical Students: Cross-Sectional Study
Source: JMIR Med Educ. 2019 May 28;5(1):e12605. doi: 10.2196/12605 (PMC6658288; doi:10.2196/12605)
Supplement: Multimedia Appendix 1 [file mededu_v5i1e12605_app1.docx]

**Table E1. Understandability and attractiveness (U&A)**

| **Item #** | **Item** | **Response Options** | **Rating** |
| --- | --- | --- | --- |
| Topic: **Organization** | | | |
| 1 | The video presents the information in a logical sequence. | Disagree=0, Agree=1 |  |
| 2 | The video provides a summary or key teaching points in the end. | Disagree=0, Agree=1 |  |
| Topic: **Visual aspects** | | | |
| 3 | The video image is clear (sharp image, adequate lighting) and uncluttered. | Disagree=0, Agree=1 |  |
| 4 | The video uses visual cues (e.g., arrows, boxes, zooming in) to draw attention to key aspects. | Disagree=0, Agree=1 |  |
| 5 | Text on the screen is easy to read. | Disagree=0, Agree=1,  No text or all text is narrated=N/A |  |
| Topic: **Auditory aspects** | | | |
| 6 | The video sound is clear and free from background noise. | Disagree=0, Agree=1, |  |
| 7 | The spoken language (instructors or voice over) is understandable (not too fast, not garbled) and can be clearly heard. | Disagree=0, Agree=1, |  |
| 8 | The used language is appropriate for medical students/ junior doctors (correct scientific or clinical terminology). | Disagree=0, Agree=1 |  |
