# Supplementary material for: YouTube Videos as a Source of Information About Immunology for Medical Students: Cross-Sectional Study
Source: JMIR Med Educ. 2019 May 28;5(1):e12605. doi: 10.2196/12605 (PMC6658288; doi:10.2196/12605)
Supplement: Multimedia Appendix 2 [file mededu_v5i1e12605_app2.docx]

**Table E2. Reliability**

| **Item #** | **Item** | **Response Options** | **Rating** |
| --- | --- | --- | --- |
| 1 | Are there valid sources sited? (from valid studies) | Disagree=0, Agree=1 |  |
| 2 | Is the information provided balanced and unbaised? | Disagree=0, Agree=1 |  |
| 3 | Are additional sources of information listed for student reference? | Disagree=0, Agree=1 |  |
| 4 | Any individuals or organizations that contribute funds, services, or material in the posted video are clearly identified in the video or video description​. | Disagree=0, Agree=1 |  |
