# Supplementary material for: YouTube Videos as a Source of Information About Immunology for Medical Students: Cross-Sectional Study
Source: JMIR Med Educ. 2019 May 28;5(1):e12605. doi: 10.2196/12605 (PMC6658288; doi:10.2196/12605)
Supplement: Multimedia Appendix 3 [file mededu_v5i1e12605_app3.docx]

**Table E3. Content and comprehensiveness (C&C): Antigen presentation**

| **Item #** | **Item** | **Response Options** | **Rating** |
| --- | --- | --- | --- |
| 1 | Explaining the name ‘Major Histocompatibility Complex’/MHC and its discovery as an important mediator in transplant graft rejection. | Fail=0, Pass=1 |  |
|  | If pass answered: good explanation of concept (Pass) or inadequate explanation of concept (Fail) | Fail=0 Pass=1 |  |
|  | Total score | 0, 1 or 2 |  |
| 2 | Showing the structure of the MHC proteins: alpha and beta subunits. | Fail=0, Pass=1 |  |
|  | If pass answered: good explanation of concept (Pass) or inadequate explanation of concept (Fail) | Fail=0, Pass=1 |  |
|  | Total score | 0, 1 or 2 |  |
| 3 | Mentioning the different HLA groups: DP, DQ, DR for class II and A, B, C for class I. | Fail=0, Pass=1 |  |
|  | If pass answered: good explanation of concept (Pass) or inadequate explanation of concept (Fail) | Fail=0, Pass=1 |  |
|  | Total score | 0, 1 or 2 |  |
| 4 | Situating the HLA/MHC genes on the human genome. | Fail=0, Pass=1 |  |
|  | If pass answered: good explanation of concept (Pass) or inadequate explanation of concept (Fail) | Fail=0, Pass=1 |  |
|  | Total score | 0, 1 or 2 |  |
| 5 | Mentioning on which cells the MHC proteins are expressed. | Fail=0, Pass=1 |  |
|  | If pass answered: good explanation of concept (Pass) or inadequate explanation of concept (Fail) | Fail=0, Pass=1 |  |
|  | Total score | 0, 1 or 2 |  |
| 6 | Explaining the interaction of MHC with the T-cell receptor and T-cell co-receptors CD8 and CD4. | Fail=0, Pass=1 |  |
|  | If pass answered: good explanation of concept (Pass) or inadequate explanation of concept (Fail) | Fail=0, Pass=1 |  |
|  | Total score | 0, 1 or 2 |  |
| 7 | Explaining the difference in antigen presentation of endogenous and exogenous antigens and the difference in immunologic response: T helper cells vs cytotoxic T cells. | Fail=0, Pass=1 |  |
|  | If pass answered: good explanation of concept (Pass) or inadequate explanation of concept (Fail) | Fail=0, Pass=1 |  |
|  | Total score | 0, 1 or 2 |  |
| 8 | MHC I: mentioning the proteasome MHC II: mentioning the phagolysosome | Fail=0, Pass=1 |  |
|  | If pass answered: good explanation of concept (Pass) or inadequate explanation of concept (Fail) | Fail=0, Pass=1 |  |
|  | Total score | 0, 1 or 2 |  |
| 9 | MHC I: mentioning the TAP1 and TAP2 molecules MHC II: mentioning the invariant chain molecule and its degradation to CLIP | Fail=0, Pass=1 |  |
|  | If pass answered: good explanation of concept (Pass) or inadequate explanation of concept (Fail) | Fail=0, Pass=1 |  |
|  | Total score | 0, 1 or 2 |  |
| 10 | MHC I: situating antigen processing in the RER MHC II: situating antigen processing in endosomes | Fail=0, Pass=1 |  |
|  | If pass answered: good explanation of concept (Pass) or inadequate explanation of concept (Fail) | Fail=0, Pass=1 |  |
|  | Total score | 0, 1 or 2 |  |
| 11 | MHC I: mentioning calnexin and/or calreticulin MHC II: mentioning HLA-DM and/or HLA-DO | Fail=0, Pass=1 |  |
|  | If pass answered: good explanation of concept (Pass) or inadequate explanation of concept (Fail) | Fail=0, Pass=1 |  |
|  | Total score | 0, 1 or 2 |  |
