# Supplementary material for: YouTube Videos as a Source of Information About Immunology for Medical Students: Cross-Sectional Study
Source: JMIR Med Educ. 2019 May 28;5(1):e12605. doi: 10.2196/12605 (PMC6658288; doi:10.2196/12605)
Supplement: Multimedia Appendix 4 [file mededu_v5i1e12605_app4.docx]

**Table E4.** **Content and comprehensiveness (C&C): Immunoglobulin gene rearrangement**

| **Item #** | **Item** | **Response Options** | **Rating** |
| --- | --- | --- | --- |
| 1 | Mentioning the purpose and the importance of antibody diversity. | Fail=0, Pass=1 |  |
|  | If pass answered: good explanation of concept (Pass) or inadequate explanation of concept (Fail) | Fail=0 Pass=1 |  |
|  | Total score | 0, 1 or 2 |  |
| 2 | Explaining the structure of a immunoglobulin: light and heavy chain. | Fail=0, Pass=1 |  |
|  | If pass answered: good explanation of concept (Pass) or inadequate explanation of concept (Fail) | Fail=0, Pass=1 |  |
|  | Total score | 0, 1 or 2 |  |
| 3 | Explaining the structure of a immunoglobulin: V (ariable) and C (constant) region. | Fail=0, Pass=1 |  |
|  | If pass answered: good explanation of concept (Pass) or inadequate explanation of concept (Fail) | Fail=0, Pass=1 |  |
|  | Total score | 0, 1 or 2 |  |
| 4 | Situating the process of VDJ recombination in the B-cell maturation. | Fail=0, Pass=1 |  |
|  | If pass answered: good explanation of concept (Pass) or inadequate explanation of concept (Fail) | Fail=0, Pass=1 |  |
|  | Total score | 0, 1 or 2 |  |
| 5 | Situating the VDJ segment in the variable region of the immunoglobulin. | Fail=0, Pass=1 |  |
|  | If pass answered: good explanation of concept (Pass) or inadequate explanation of concept (Fail) | Fail=0, Pass=1 |  |
|  | Total score | 0, 1 or 2 |  |
| 6 | Explaining recombination in the heavy chain: V(ariable), D(iversity), J(oining) and C(onstant) segments. | Fail=0, Pass=1 |  |
|  | If pass answered: good explanation of concept (Pass) or inadequate explanation of concept (Fail) | Fail=0, Pass=1 |  |
|  | Total score | 0, 1 or 2 |  |
| 7 | Explaining recombination of the light chain: VJ recombination. | Fail=0, Pass=1 |  |
|  | If pass answered: good explanation of concept (Pass) or inadequate explanation of concept (Fail) | Fail=0, Pass=1 |  |
|  | Total score | 0, 1 or 2 |  |
| 8 | Explaining the $\lambda$light chain and the $\kappa$ light chain. | Fail=0, Pass=1 |  |
|  | If pass answered: good explanation of concept (Pass) or inadequate explanation of concept (Fail) | Fail=0, Pass=1 |  |
|  | Total score | 0, 1 or 2 |  |
| 9 | Explaining the function of RSS. | Fail=0, Pass=1 |  |
|  | If pass answered: good explanation of concept (Pass) or inadequate explanation of concept (Fail) | Fail=0, Pass=1 |  |
|  | Total score | 0, 1 or 2 |  |
| 10. | Explaining the 23/12 rule. |  |  |
|  | If pass answered: good explanation of concept (Pass) or inadequate explanation of concept (Fail) | Fail=0, Pass=1 |  |
|  | Total score | 0, 1 or 2 |  |
| 11 | Explaining the functioning on the proteins RAG1/2. | Fail=0, Pass=1 |  |
|  | If pass answered: good explanation of concept (Pass) or inadequate explanation of concept (Fail) | Fail=0, Pass=1 |  |
|  | Total score | 0, 1 or 2 |  |
| 12 | Explaining the functioning on the protein Tdt. | Fail=0, Pass=1 |  |
|  | If pass answered: good explanation of concept (Pass) or inadequate explanation of concept (Fail) | Fail=0, Pass=1 |  |
|  | Total score | 0, 1 or 2 |  |
| 13 | Mentioning the fact that during recombination new nucleotides can be added (by Tdt) which causes more diversity. | Fail=0, Pass=1 |  |
|  | If pass answered: good explanation of concept (Pass) or inadequate explanation of concept (Fail) | Fail=0, Pass=1 |  |
|  | Total score | 0, 1 or 2 |  |
