# Supplementary material for: YouTube Videos as a Source of Information About Immunology for Medical Students: Cross-Sectional Study
Source: JMIR Med Educ. 2019 May 28;5(1):e12605. doi: 10.2196/12605 (PMC6658288; doi:10.2196/12605)
Supplement: Multimedia Appendix 5 [file mededu_v5i1e12605_app5.docx]

**Table E5. Global Quality Score**

| **Item #** | **Item** |
| --- | --- |
| 1 | Poor quality, poor flow, most information missing, not helpful for students. |
| 2 | Generally poor, some information given but of limited use for students. |
| 3 | Moderate quality, some import information is adequately discussed, but important topics are missing. Somewhat useful to students. |
| 4 | Good quality flow, most relevant information is covered, useful for students. |
| 5 | Excellent quality and flow, very useful for students. |
