# Supplementary material for: YouTube Videos as a Source of Information About Immunology for Medical Students: Cross-Sectional Study
Source: JMIR Med Educ. 2019 May 28;5(1):e12605. doi: 10.2196/12605 (PMC6658288; doi:10.2196/12605)
Supplement: Multimedia Appendix 6 [file mededu_v5i1e12605_app6.docx]

**Table E6. Audience interaction parameters**

| **Variable** | **YouTube Entertainment** | **YouTube Biology** | **Antigen presentation** | **Immunoglobulin gene rearrangement** | ***P*** |
| --- | --- | --- | --- | --- | --- |
| **Video number, n** | 100 | 100 | 82 | 70 |  |
| **Audience interaction parameters** |  |  |  |  |  |
| Views^a^ | 11192 (9192-13191) | 171 (99.2-243) | 53.6 (20. 5-86.7) | 22.5 (9.2-35.8) | **<.001** |
| Likes^a^ | 347 (281-413) | 4.8 (3.1-6.5) | 0.4 (0.08-0.8) | 0.2 (0.06-0.3) | **<.001** |
| Dislikes | 19641 (12224-27058) | 138 (87.1-189) | 10.35 (4.05-16.66) | 4.28 (1.84-6.72) | **<.001** |
| Like ratio | 93.7 (92.1-95.2) | 95.9 (94.9-96.9) | 94.2 (90.8-97.5) | 85.9 (77.3-94.4) | **.001** |
| Comments | 59416 (47714-71119) | 700 (303-1097) | 30.5 (6.4-54.5) | 14.1 (6.9-21.2) | **<.001** |
| Days since upload | 52.3 (47.0-57.6) | 400 (362-439) | 1241 (1031-1452) | 1241 (1042-1440) | **<.001** |
| View ratio^a^ | 364 (247-482) | 1.1 (0.3-1.9) | 0.04 (0.009-0.07) | 0.01 (0.007-0.02) | **<.001** |
| Length, sec | 960 (813-1108) | 818 (645-990) | 599 (467-731) | 434 (349-519) | **<.001** |
| VPI^a^ | 340 (231-449) | 1.0 (0.2-1.8) | 0.04 (0.009-0.07) | 0.02 (0.007-0.02) | **<.001** |
| VPI: video power index.  Values are presented as mean (95% CI) or n (%).  P<0.05 was considered significant.  a These factors were divided by 1,000 | | | | | |
