# Supplementary material for: YouTube Videos as a Source of Information About Immunology for Medical Students: Cross-Sectional Study
Source: JMIR Med Educ. 2019 May 28;5(1):e12605. doi: 10.2196/12605 (PMC6658288; doi:10.2196/12605)
Supplement: Multimedia Appendix 7 [file mededu_v5i1e12605_app7.docx]

**Table E7. Audience interaction parameters, pairwise comparison**

|  | ***P*** |  |  |  |  |  |
| --- | --- | --- | --- | --- | --- | --- |
|  | **Group 1-2** | **Group 1-3** | **Group 1-4** | **Group 2-3** | **Group 2-4** | **Group 3-4** |
| **Audience interaction parameters** |  |  |  |  |  |  |
| Views | **<0.05** | **<0.05** | **<0.05** | NS | NS | NS |
| Likes | **<0.05** | **<0.05** | **<0.05** | NS | NS | NS |
| Dislikes | **<0.05** | **<0.05** | **<0.05** | NS | NS | NS |
| Like ratio | NS | NS | **<0.05** | NS | **<0.05** | **<0.05** |
| Comments | **<0.05** | **<0.05** | **<0.05** | NS | NS | NS |
| Days since upload | **<0.05** | **<0.05** | **<0.05** | **<0.05** | **<0.05** | NS |
| View ratio | **<0.05** | **<0.05** | **<0.05** | NS | NS | NS |
| Length, sec | NS | **<0.05** | **<0.05** | NS | **<0.05** | NS |
| VPI | **<0.05** | **<0.05** | **<0.05** | NS | NS | NS |
| GQS: global quality score  Pairs indicated by P<0.05 were significantly different in REGWq post hoc analysis<, while pairs indicated by NS were not. | | | | | | |
