# Supplementary material for: YouTube Videos as a Source of Information About Immunology for Medical Students: Cross-Sectional Study
Source: JMIR Med Educ. 2019 May 28;5(1):e12605. doi: 10.2196/12605 (PMC6658288; doi:10.2196/12605)
Supplement: Multimedia Appendix 8 [file mededu_v5i1e12605_app8.docx]

**Table E8. Antigen presentation videos: categorized by source, pairwise comparison**

|  | ***P*** |  |  |
| --- | --- | --- | --- |
|  | **Group 1-2** | **Group 1-3** | **Group 2-3** |
| **Audience interaction parameters** |  |  |  |
| Views | **.006** | .07 | 1 |
| Likes | **.007** | .44 | 1 |
| Dislikes | **.01** | .15 | 1 |
| Comments | **.001** | .39 | 1 |
| Days since upload | 1 | **.02** | .06 |
| View ratio | **.001** | .22 | 1. |
| Length, sec | .21 | .24 | **.004** |
| VPI | **.005** | .28 | 1 |
| **Content** |  |  |  |
| Reliability | .16 | **.01** | .24 |
| C&C | .08 | 1 | .30 |
| GQS | **.009** | 1 | **.02** |
| C&C: content and comprehensiveness; GQS: global quality score, VPI: video power index.  P<.05 was considered significant. | | | |
