# Supplementary material for: YouTube Videos as a Source of Information About Immunology for Medical Students: Cross-Sectional Study
Source: JMIR Med Educ. 2019 May 28;5(1):e12605. doi: 10.2196/12605 (PMC6658288; doi:10.2196/12605)
Supplement: Multimedia Appendix 9 [file mededu_v5i1e12605_app9.docx]

**Table E9. Immunoglobulin gene rearrangement videos: categorized by source, pairwise comparison**

|  | ***P*** |  |  |
| --- | --- | --- | --- |
|  | **Group 1-2** | **Group 1-3** | **Group 2-3** |
| **Audience interaction parameters** |  |  |  |
| Days since upload | **.03** | **.02** | 1 |
| Length, sec | 1 | .15 | **<.001** |
| **Content** |  |  |  |
| Reliability | **.002** | .45 | **.03** |
| GQS | 1 | .65 | **.02** |
| GQS: global quality score.  P<.05 was considered significant. | | | |
