# Supplementary material for: YouTube Videos as a Source of Information About Immunology for Medical Students: Cross-Sectional Study
Source: JMIR Med Educ. 2019 May 28;5(1):e12605. doi: 10.2196/12605 (PMC6658288; doi:10.2196/12605)
Supplement: Multimedia Appendix 10 [file mededu_v5i1e12605_app10.docx]

**Table E8. Correlations between video scoring systems in the antigen presentation group.**

|  | VPI | Reliability | C&C | GQS | U&A | Subjective |
| --- | --- | --- | --- | --- | --- | --- |
| VPI | 1 |  |  |  |  |  |
|  |  |  |  |  |  |  |
| Reliability | .02 | 1 |  |  |  |  |
|  | (.86) |  |  |  |  |  |
| C&C | -.10 | .12 | 1 |  |  |  |
|  | (.39) | (.30) |  |  |  |  |
| GQS | .07 | .14 | .84 | 1 |  |  |
|  | (.54) | (.21) | (<.001) |  |  |  |
| U&A | .25 | .04 | .14 | .29 | 1 |  |
|  | (.03) | (.73) | (.20) | (.01) |  |  |
| Subjective | .18 | .21 | .23 | .38 | .49 | 1 |
|  | (.13) | (.06) | (.04) | (<.001) | (<.001) |  |
| C&C: content and comprehensiveness; GQS: global quality score; U&A: understandability and attractiveness, VPI: video power index.  P<0.05 was considered significant.  Evaluating the correlations between the different scoring systems in the antigen presentation cohort, a positive correlation was found between following pairs: 22,6% between subjective score and C&C (p=0.04), 25,0% between VPI and U&A (p=0.03), 28,7% between GQS and U&A (p=0.01), 38,0% between GQS and subjective score (p<.001), 49,0% between subjective score and U&A (p<.001), and 83,5% between GQS and C&C (p<.001). | | | | | | |
