# Supplementary material for: YouTube Videos as a Source of Information About Immunology for Medical Students: Cross-Sectional Study
Source: JMIR Med Educ. 2019 May 28;5(1):e12605. doi: 10.2196/12605 (PMC6658288; doi:10.2196/12605)
Supplement: Multimedia Appendix 11 [file mededu_v5i1e12605_app11.docx]

**Table E9. Correlations between video scoring systems in the immunoglobulin gene rearrangement group.**

|  | VPI | Reliability | C&C | GQS | U&A | Subjective |
| --- | --- | --- | --- | --- | --- | --- |
| VPI | 1 |  |  |  |  |  |
|  |  |  |  |  |  |  |
| Reliability | .28 | 1 |  |  |  |  |
|  | (.05) |  |  |  |  |  |
| C&C | .26 | 0.07 | 1 |  |  |  |
|  | (.07) | (0.58) |  |  |  |  |
| GQS | .14 | .21 | .78 | 1 |  |  |
|  | (.32) | (.08) | (<.001) |  |  |  |
| U&A | .28 | .33 | .37 | .54 | 1 |  |
|  | (.05) | (.005) | (.002) | (<.001) |  |  |
| Subjective | .38 | .38 | .35 | .56 | .59 | 1 |
|  | (.007) | (.001) | (.003) | (<.001) | (<.001) |  |
| C&C: content and comprehensiveness; GQS: global quality score; U&A: understandability and attractiveness, VPI: video power index.  P<0.05 was considered significant.  Evaluating the correlations between the different scoring systems in the immunoglobulin gene rearrangement cohort, a positive correlation was found between following pairs: 27,9% between VPI and U&A (p=0.05), 28,1% between VPI and reliability (p=0.05), 33,3% between reliability and U&A (p=0.01), 34,9% between subjective score and C&C (p=0.003), 36,8% between C&C and U&A (p=0.002), 37,6% between VPI and subjective score (p=0.007), 38,2% between subjective score and reliability score (p=0.001), 54,1% between GQS and U&A (p<.001), 56,0% between GQS and subjective score (p<.001), 59,2% between subjective score and U&A (p<.001), and 77,8% between GQS and C&C (p<.001). | | | | | | |
